# Copine-7 binds to the cell surface receptor, nucleolin, and regulates ciliogenesis and Dspp expression during odontoblast differentiation

**DOI:** 10.1038/s41598-017-11641-y

**Published:** 2017-09-12

**Authors:** You-Mi Seo, Su-Jin Park, Hye-Kyung Lee, Joo-Cheol Park

**Affiliations:** Department of Oral Histology-Developmental Biology, School of Dentistry and Dental Research Institute, Seoul National University, Seoul, Republic of Korea

## Abstract

Tooth development is a progressive process regulated by interactions between epithelial and mesenchymal tissues. Our previous studies showed that copine-7 (Cpne7), a dental epithelium-derived protein, is a signalling molecule that is secreted by preameloblasts and regulates the differentiation of preodontoblasts into odontoblasts. However, the mechanisms involved in the translocation of Cpne7 from preameloblasts to preodontoblasts and the functions of Cpne7 during odontogenesis are poorly understood. Here, we showed that the internalization of Cpne7 was mediated primarily by caveolae. This process was initiated by Cpne7 binding to the cell surface protein, nucleolin. Treatment with recombinant Cpne7 protein (rCpne7) in human dental pulp cells (hDPCs) caused an increase in the number of ciliated cells. The expression level of cilium components, Ift88 and Kif3a, and Dspp were increased by rCpne7. Treatment with Ift88 siRNA in hDPCs and MDPC-23 cells significantly down-regulated the expression of Dspp, an odontoblastic differentiation marker gene. Furthermore, the treatment with nucleolin siRNA in MDPC-23 cells decreased the expression of Dmp1, Dspp, and cilium components. Our findings suggested that the binding of Cpne7 with its receptor, nucleolin, has an important function involving Cpne7 internalization into preodontoblasts and regulation of Dspp expression through ciliogenesis during odontoblast differentiation.

## Introduction

Tooth development is a consequence of programmed, sequential, and reciprocal communications between the dental epithelium and mesenchyme, which is also mediated by specific temporal-spatial expression of a series of genes^1^. Interactions between the ectodermal tissue and underlying mesenchymal tissue form the basis of the mechanism that regulates tooth development. Epithelial and mesenchymal cells differentiate into ameloblasts and odontoblasts, respectively, during crown formation^2^. In 1887, Von Brunn suggested that odontoblasts differentiated only in the presence of the enamel epithelia^3^. This study reported that epithelial signals induced in the mesenchyme led to subsequent odontoblast differentiation and dentin formation.

Based on the concept of epithelial-mesenchymal interactions during odontogenesis, we previously investigated the effects of preameloblast-conditioned medium (PA-CM) on the odontogenic differentiation of human dental pulp cells (hDPCs). Our previous report showed that dental epithelium-derived factors in PA-CM induced odontogenic differentiation of hDPCs. Among those secreted dental epithelium-derived factors, copine-7 (Cpne7) was expressed in preameloblasts and secreted extracellularly during ameloblast differentiation. After secretion, the Cpne7 protein was translocated to differentiating odontoblasts and to induce the expression of Dspp, which is a major component of the non-collagenous dentin extracellular matrix and odontoblast differentiation *in vitro* and *in vivo* ^4^. However, the mechanism involved in the translocation of Cpne7 from preameloblasts to preodontoblasts is poorly understood.

Cpne is a ubiquitous family of calcium-dependent phospholipid-binding proteins that is evolutionally conserved in animals, plants, and protists. Nine Cpne genes have been identified. Cpnes have conserved features consisting of two C2 domains (C2A and C2B) and the von Willebrand factor A (vWA) domain. The C2 domains of Cpnes were originally identified in conventional protein kinase C (PKC) and were involved in calcium influx^5^. The vWA domain mediates protein-protein interactions^6^. Although the function of Cpnes remains unclear, some of the biological roles of several Cpnes are known. In mammals, Cpnes are widely expressed throughout different tissues, including the brain, heart, lung, liver, and kidney^7^. Cpne1, 2, and 3 are expressed in all normal tissues. Cpnes4-7 show more limited expression. Cpne4 is found in brain, heart, and prostate glands, and Cpne6 is brain specific. Cpne7 is expressed in foetal brain, thymus, and testis^8^. All of the Cpnes exhibit calcium–dependent translocation to the plasma membrane, and Cpne 1, 2, 3, and 7 also translocate to the nucleus^9^. Cpne6 links activity-triggered calcium signals to spine structural plasticity that is necessary for learning and memory^10^. However, there have been few studies of the functions of Cpne7 besides our previous report that Cpne7, a diffusing signalling molecule, is a regulator of the differentiation of mesenchymal cells into odontoblasts.

The primary cilium has been found in almost every eukaryotic cell type as a non-motile antenna emerging from the cell and extending into the extracellular space^11^. In many tissues, primary cilia are essential for sensing mechanical, biochemical or light signals^12, 13^. Mutations in genes encoding cilium components involved in major human genetic diseases including developmental disorders, dysfunctions of the reproductive system, airway disease, cystic disorders of the kidney, liver, and pancreas, defects in vision, smell and hearing, and oncogenesis^14^. Consequently, the primary cilium plays a fundamental role in cellular physiology and development including tooth formation, bone formation, and nerve formation^15–17^. Among the Cpne family, Cpne6 was identified in ciliary bodies^18^. However, the molecular mechanisms responsible for inducing cilianogenesis during odontogenesis remain unclear.

In this study, we showed that the internalization of Cpne7, an epithelium-derived factor, into the odontoblasts is a caveolae-dependent, receptor-mediated event and that Cpne7 regulated odontoblast differentiation through the formation of primary cilia. Three major steps highlighted in this study involved: nucleolin functioning as a cell surface receptor in Cpne7 endocytosis, Cpne7 regulation of ciliogenesis, and Cpne7 regulation of Dspp expression via Ift88, one of the ciliary components.

## Results

### Endocytosed Cpne7 is localized in the cytoplasm and nucleus

**Figure 1:**
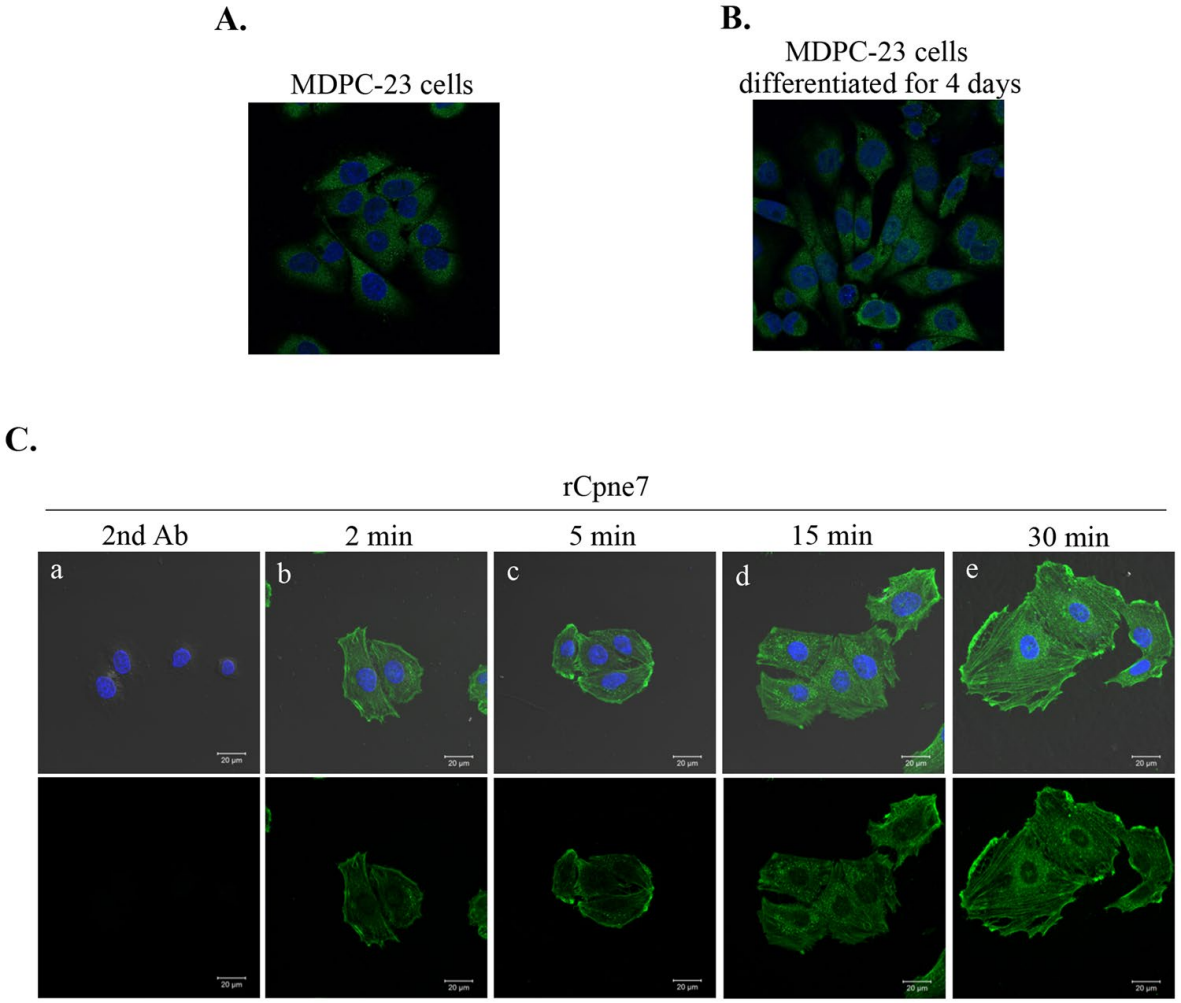
Intracellular distribution of Cpne7 in odontoblastic MDPC-23 cells. (**A,B**) Cellular localization of Cpne7 was detected by immunofluorescence before (**A**) or after odontoblastic differentiation (**B**) of MDPC-23 cells. (**C**) The time course confocal images of internalized Cpne7 were detected using anti-Flag antibody in MDPC-23 cells after rCpne7 treatment for 2, 5, 15, and 30 min. Each data are representative of two or three independently performed experiments. Scale bars, 20 μm.

The cellular localization of endogenous Cpne7 was evaluated in odontoblastic MDPC-23 cells. Cpne7 was localized in the cytoplasm and nucleus in odontoblastic cells (Fig. 1A,B). Nuclear-localized Cpne7 was especially increased during odontoblast differentiation (Fig. 1B). To further validate the internalization process, MDPC-23 cells were treated for 2, 5, 15 and 30 min with recombinant Flag-Cpne7 protein (rCpne7). Analyses by confocal microscopy showed that Cpne7 was endocytosed and transported to the perinuclear region by 30 min (Fig. 1Ce). However, the secondary antibody treated-group, which served as the control, was not internalized (Fig. 1Ca).

### Cellular uptake of Cpne7 occurs through caveolae-dependent receptor-mediated endocytosis

**Figure 2:**
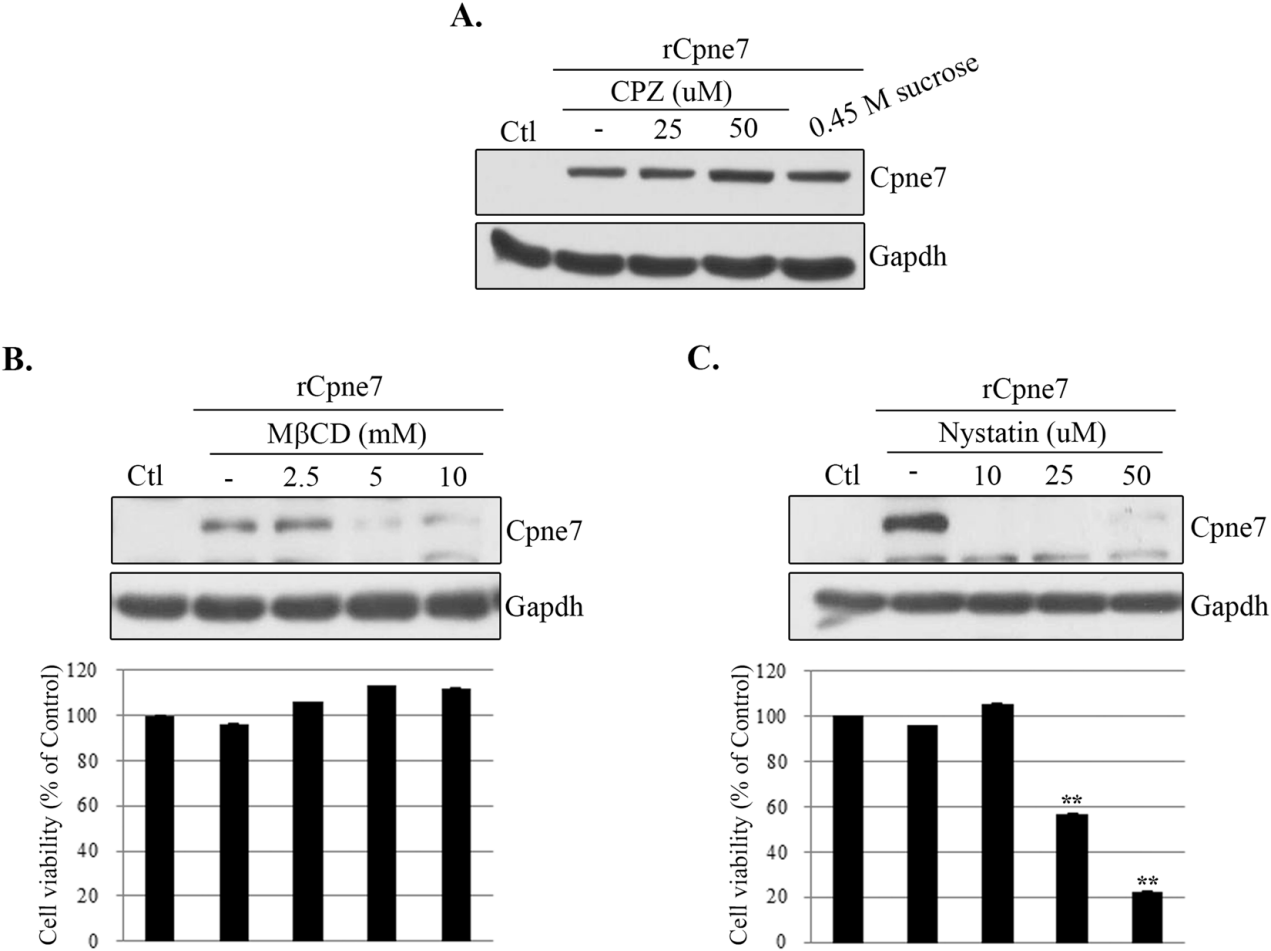
Effects of receptor-mediated endocytic inhibitors on Cpne7 endocytosis in odontoblastic MDPC-23 cells. MDPC-23 cells were pre-treated with varying concentrations of chlorpromazine (CPZ; 25 and 50 μM) (**A**), 0.45 M sucrose (**A**), methyl-beta-cyclodextrin (MβCD; 2.5, 5, and 10 mM) (**B**) and nystatin (10, 25, and 50 μM) (**C**) for 1 h before rCpne7 treatment. Internalized Cpne7 was detected by western blotting. The effects of drug treatment on cell viability were assessed using the MTT assay. All values represent the mean ± standard deviation of triplicate experiments. **P < 0.001 compared with the control.

Receptor-mediated endocytosis is a process by which cells absorb metabolites, hormones, other proteins, and, in some cases, viruses by inward budding of plasma membrane vesicles containing proteins with receptor sites specific to the molecules being absorbed. To elucidate the characteristics of the endocytic pathway of Cpne7, we tested the effect of drugs that inhibit clathrin or caveolae-mediated endocytosis. Use of chlorpromazine and 0.45M sucrose, which inhibits clathrin-mediated endocytosis, did not have any effect (Fig. 2A). Pre-treatment of the cells with inhibitors of lipid raft/caveolae-dependent endocytosis, methyl-β-cyclodextrin or nystatin, significantly inhibited cellular uptake of Cpne7 (Fig. 2B,C). Possible drug-induced cytotoxic effects were assessed by MTT cell viability assays (Fig. 2B,C). These results indicated that Cpne7 uptake into MDPC-23 cells could be mediated via the lipid raft/caveolae pathway.

### Nucleolin is a cell surface receptor of Cpne7

**Figure 3:**
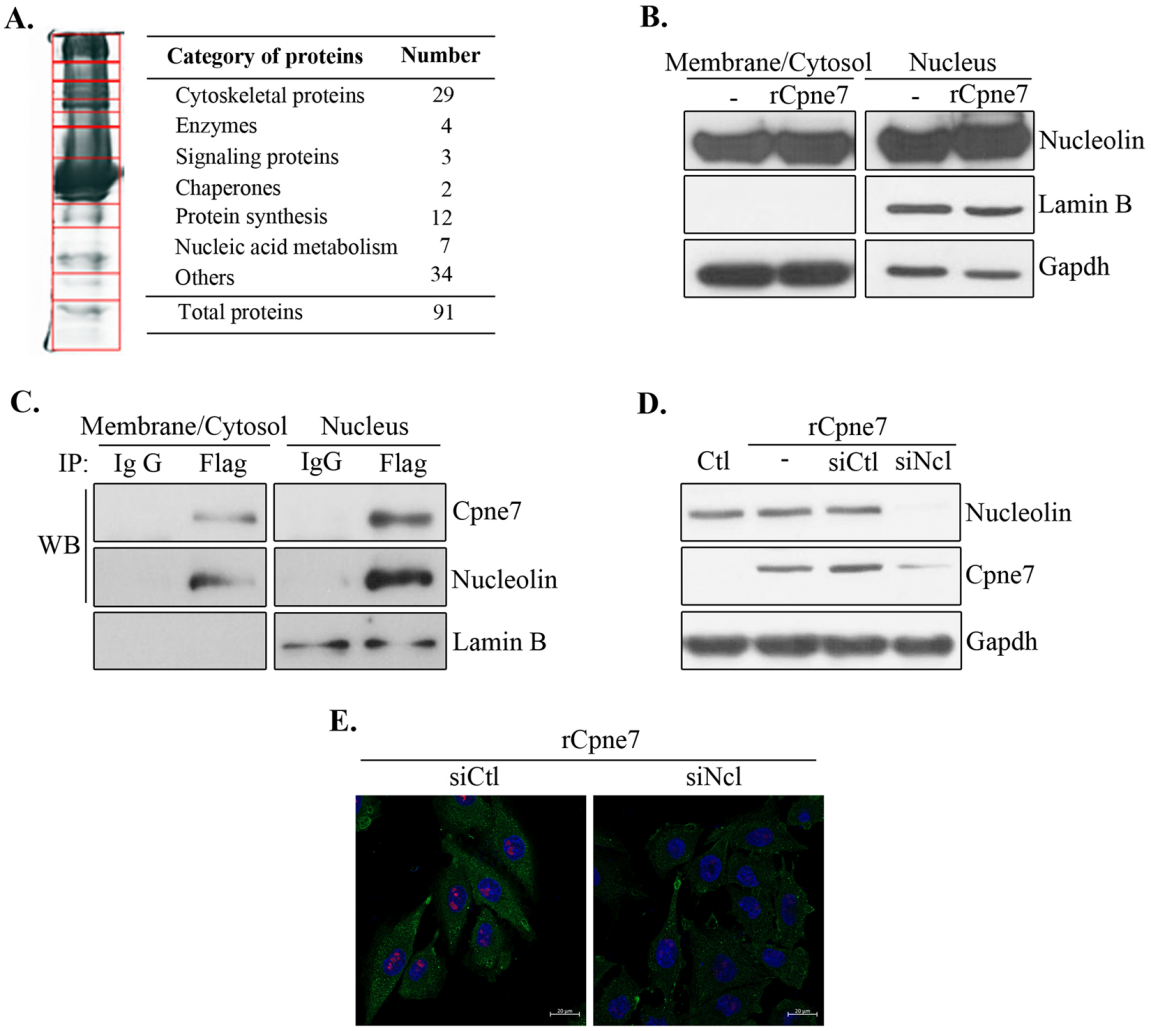
Identification of a receptor, nucleolin which mediates Cpne7 endocytosis. (**A**) Total lysates obtained from Flag-Cpne7-overexpressed MDPC-23 cells were immunoprecipitated with anti-Flag antibody. The samples were subjected to SDS-PAGE followed by Coomassie blue staining. Eleven compartments were excised from the gel and analysed by tendem mass spectrometry. (**B**) The cell lysates were separated into membrane/cytoplasmic and nuclear fractions and then analysed for nucleolin proteins by western blotting. (**C**) The interaction with Cpne7 and nucleolin was analysed by co-immunoprecipitation. Lamin B served as nucleus fractionation control. (**D,E**) Endocytosis of Cpne7 in MDPC-23 cells after treatment with nucleolin siRNA. MDPC-23 cells were pre-treated with nucleolin siRNA for 1 h before Cpne7 protein (rCpne7) treatment. Internalized Cpne7 was detected by western blotting (**D**) or immunostaining (**E**). Each data are representative of two or three independently performed experiments. siCtl, control siRNA; siNcl, nucleolin siRNA; Gapdh, glyceraldehyde 3-phosphate dehydrogenase.

Previous results indicated that Cpne7 underwent receptor-mediated endocytosis. To identify the receptor of Cpne7 in preodontoblasts, total lysates were immunoprecipitated with Flag antibody in Flag-Cpne7 transfected-MDPC-23 cells, and then resolved by SDS-PAGE. The individual compartments were excised from the gel, and the proteins were analysed by tandem mass spectrometry (LC-MS/MS; Fig. 3A). Most of the proteins were cytoskeletal components implicated in the maintenance and the motility of microvilli. Only two proteins (nucleolin and Slitrk1) of the Cpne7-interacting proteins were localized to the cellular membrane. Surface nucleolin, has recently attracted increasing attention as an important cell receptor for numerous ligands derived from various sources^19^. Nucleolin also mediates internalization of endostatin^20^, LPS^21^, DNA nanoparticles^22^, and lactoferrin^23^. To confirm that Cpne7 binds specifically to nucleolin, we performed immunoprecipitation using anti-Flag antibody. Nucleolin interacted with Cpne7 in the membrane/cytosol and nuclear fractions (Fig. 3C) and was also expressed in the membrane/cytosol and nuclear fractions (Fig. 3B).

To confirm that nucleolin is a receptor that mediates endocytosis of Cpne7, we treated cells with nucleolin siRNA before rCpne7 treatment and analysed Cpne7 internalization by western blotting and immunostaining. Figure 3D and E show that Cpne7 internalization was abrogated with nucleolin siRNA. These results confirmed that nucleolin mediated Cpne7 endocytosis into preodontoblasts.

### Cpne7 endocytosis is dependent on calcium

**Figure 4:**
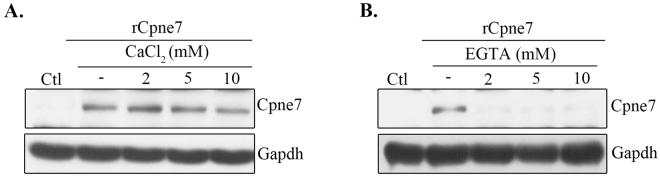
Calcium dependence of Cpne7 endocytosis. Endocytosis of Cpne7 in MDPC-23 cells after treatment with CaCl_2_ (**A**) or EGTA (**B**). MDPC-23 cells were pre-treated with varying concentrations of CaCl_2_ or EGTA for 1 h before rCpne7 treatment. The internalized Cpne7 was detected by western blotting. Each data are representative of three independently performed experiments. Gapdh, glyceraldehyde 3-phosphate dehydrogenase.

In many cells, endocytosis is Ca^2+^-dependent^24^. The molecular machinery mediating Ca^2+^-dependent endocytosis was best characterized in the context of synaptic vesicle recycling^25, 26^. The Ca^2+^ influx through Ca^2+^ channels triggering endocytosis is also well established in non-excitable cells, such as oocytes^27^. We therefore examined whether Ca^2+^ entry was responsible for Cpne7 endocytosis. Treatment with CaCl_2_ did not alter the amount of Cpne7 endocytosis (Fig. 4A), and Ca^2+^ did not affect Cpne7 endocytosis because the MDPC-23 cell culture medium, DMEM, contained 1.8 mM CaCl_2_. However, EGTA, Ca^2+^ chelator, prevented Cpne7 endocytosis (Fig. 4B), suggesting that Cpne7 endocytosis was dependent on Ca^2+^.

### Cpne7 regulates ciliogenesis during odontoblast differentiation

**Figure 5:**
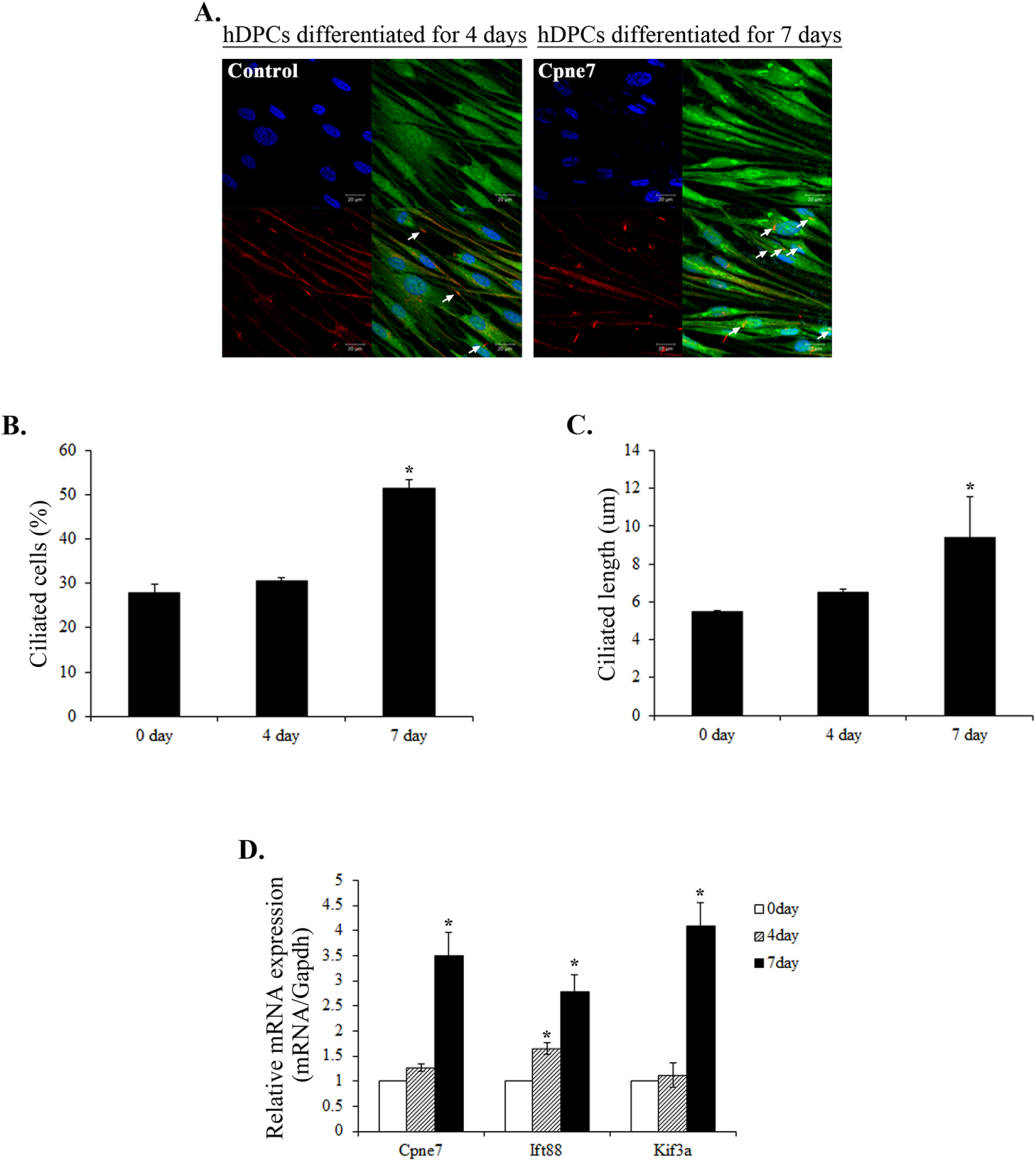
Expression of Cpne7, primary cilia and cilium components during the differentiation of hDPCs. (**A**) Primary cilia were detected with acetylated α-tubulin (red), Cpne7 (green) immunostaining, and counterstained with DAPI following 4 and 7 days of differentiation in defined odontogenic inductive medium. Arrows indicate α-tubulin immunostained primary cilia. (**B**) Counterstaining with DAPI was used to calculate the percentage of primary cilia in the hDPCs. (**C**) Confocal images of cilia were randomly captured and cilium length was analysed using Olympus software. (**D**) Expression of Cpne7 and cilium components, Ift88 and Kif3a in hDPCs during differentiation was assessed using reverse transcription-quantitative polymerase chain reaction analysis. The data are presented as the mean ± standard deviation of three independently performed experiments. *P < 0.05 compared with day 0. Scale bars, 20 μm.

**Figure 6:**
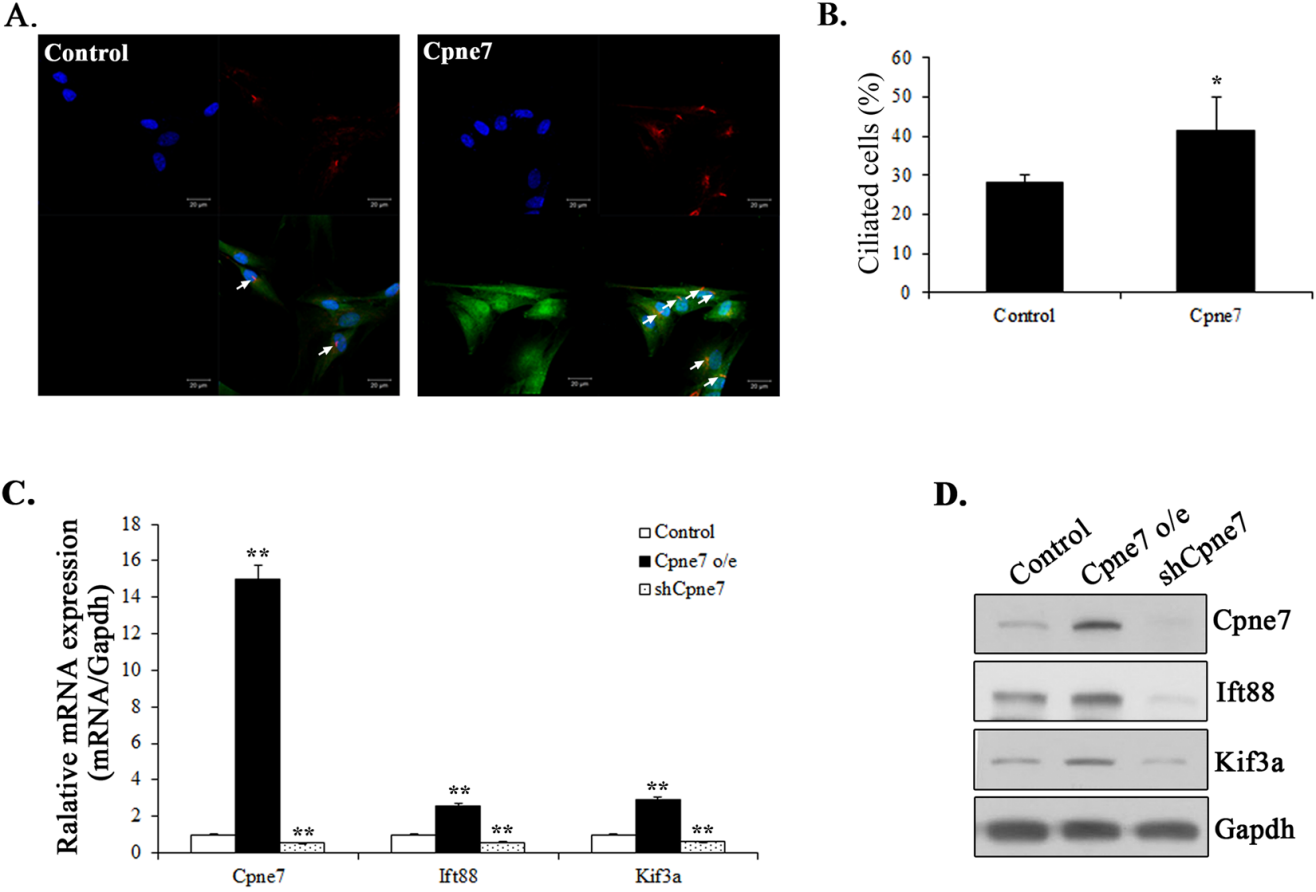
Effect of Cpne7 on ciliogenesis in hDPCs. (**A**) Human DPCs were immunostained with acetylated α-tubulin antibody (red) in the presence or absence of Cpne7 (green) and the presence of cilia was analysed by confocal microscopy. (**B**) The percentage of cells with cilia was counted for the indicated group. (**C,D**) The expression levels of cilium components by Cpne7 were examined by quantitative real-time polymerase chain reaction (**C**) and western blotting (**D**) after Cpne7 overexpression or shRNA treatment for 2 days. All values represent the mean ± standard deviation of three independently performed experiments. **P < 0.001, *P < 0.05 compared with control. Scale bars, 20 μm.

Primary cilia play an essential role not only in the initiation of both osteogenic and adipogenic differentiation, but also in the maintenance of the phenotype of differentiated cells^28^. To assess whether the presence of primary cilium components had any physiological relevance in odontoblast differentiation, we examined the number of primary cilia and the expression of cilium components during odontoblast differentiation using immunofluorescence microscopy and real-time PCR. The basal body and axoneme of primary cilium can be stained with γ-tubulin and acetylated α-tubulin antibody, respectively. We detected the existence of primary cilia with anti-acetylated α-tubulin antibody. The number of ciliated cells and the cilium length gradually increased following odontoblast differentiation (Fig. 5A–C). In addition, the mRNA of Cpne7 and cilium components, Ift88 and Kif3a, respectively, increased progressively during hDPC differentiation (Fig. 5D). To examine whether Cpne7 regulated ciliogenesis during odontogenesis, we performed immunofluorescence microscopy of cilium components in hDPCs after transfection with a Cpne7 construct. Cpne7 increased the number of ciliated cells (Fig. 6A,B). To confirm this observation, we also investigated the mRNA and protein levels of cilium components after Cpne7 overexpression or shRNA treatment. As expected, the expression level of the cilium components, Ift88 and Kif3a, was increased after Cpne7 treatment and was inhibited by Cpne7 shRNA treatment in hDPCs (Fig. 6C,D) and MDPC23 cells (Supplementary Fig. 1A,B). Taken together, these results suggested that Cpne7 affected ciliogenesis during odontoblast differentiation.

### Cpne7 regulates Dspp expression via ciliogenesis in odontoblasts

**Figure 7:**
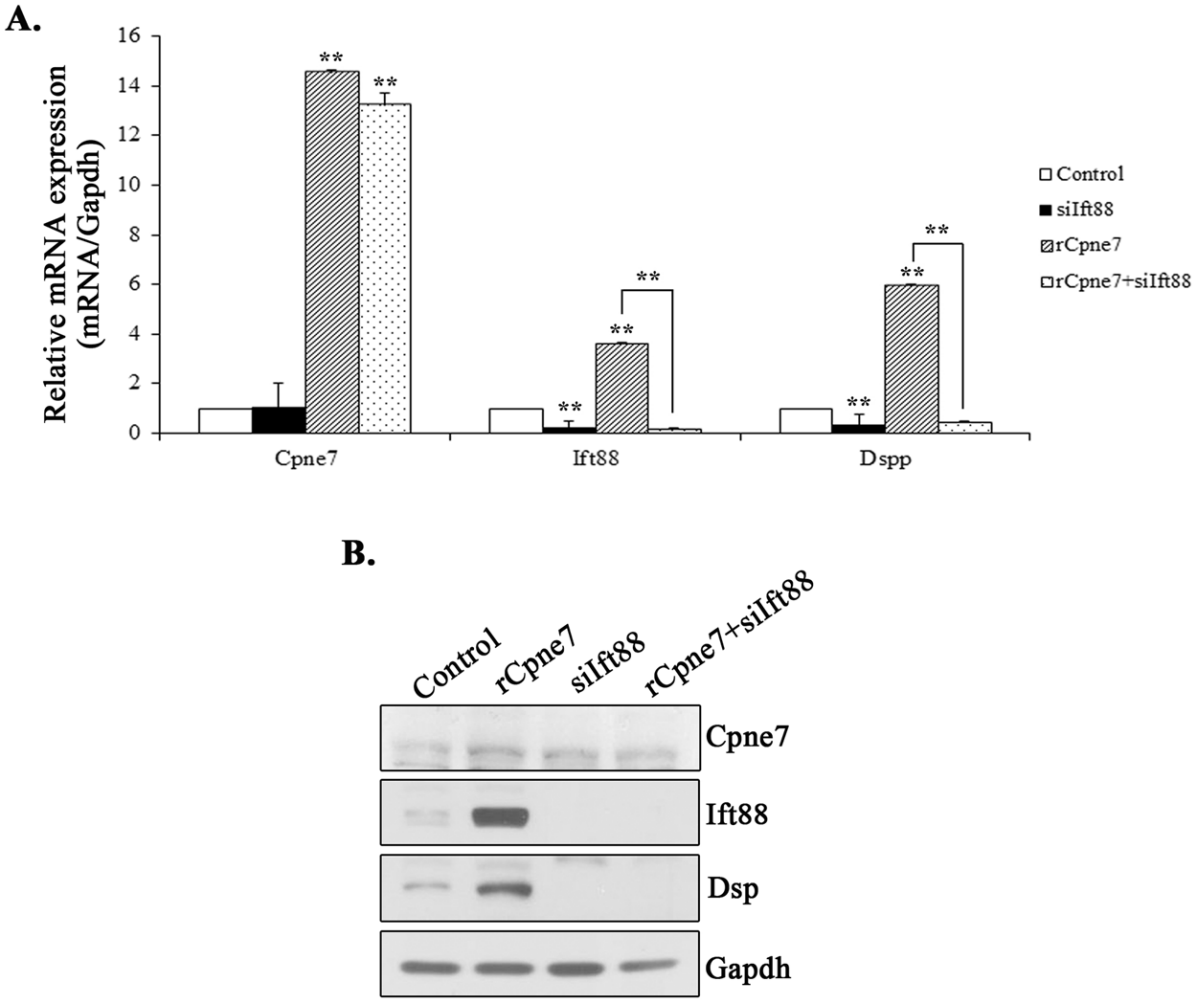
Effect of Ift88 knockdown on Dspp expression in hDPCs. (**A,B**) Expression levels of Dspp were examined by the quantitative real-time polymerase chain reaction and western blotting after Ift88 siRNA treatment for 2 days. All values represent the mean ± standard deviation of three independently performed experiments. **P < 0.001 compared with the control.

We previously reported that Cpne7 controlled Dspp expression during odontoblast differentiation^4^. The results of Fig. 6 show that Cpne7 regulated ciliogenesis during odontoblast differentiation. To evaluate whether primary cilia and cilium components mediated the regulation of Dspp expression by Cpne7, we investigated the expression of Dspp after treatment of rCpne7 in the presence or absence of Ift88 siRNA in hDPCs and MDPC-23 cells using real-time PCR and western blot analyses. The presence of rCpne7 increased the transcription of Ift88 and Dspp, whereas hDPCs and MDPC-23 cells transfected with Ift88 siRNA had inhibited Dspp expression (Fig. 7A,B, Supplementary Fig. 2A,B). Together, these results suggested that Cpne7 secreted from ameloblasts affected ciliogenesis and odontogenesis by controlling the expression of cilium components and Dspp.

### Expression of ciliary components and Dspp is induced by binding of Cpne7 and its membrane receptor, nucleolin

**Figure 8:**
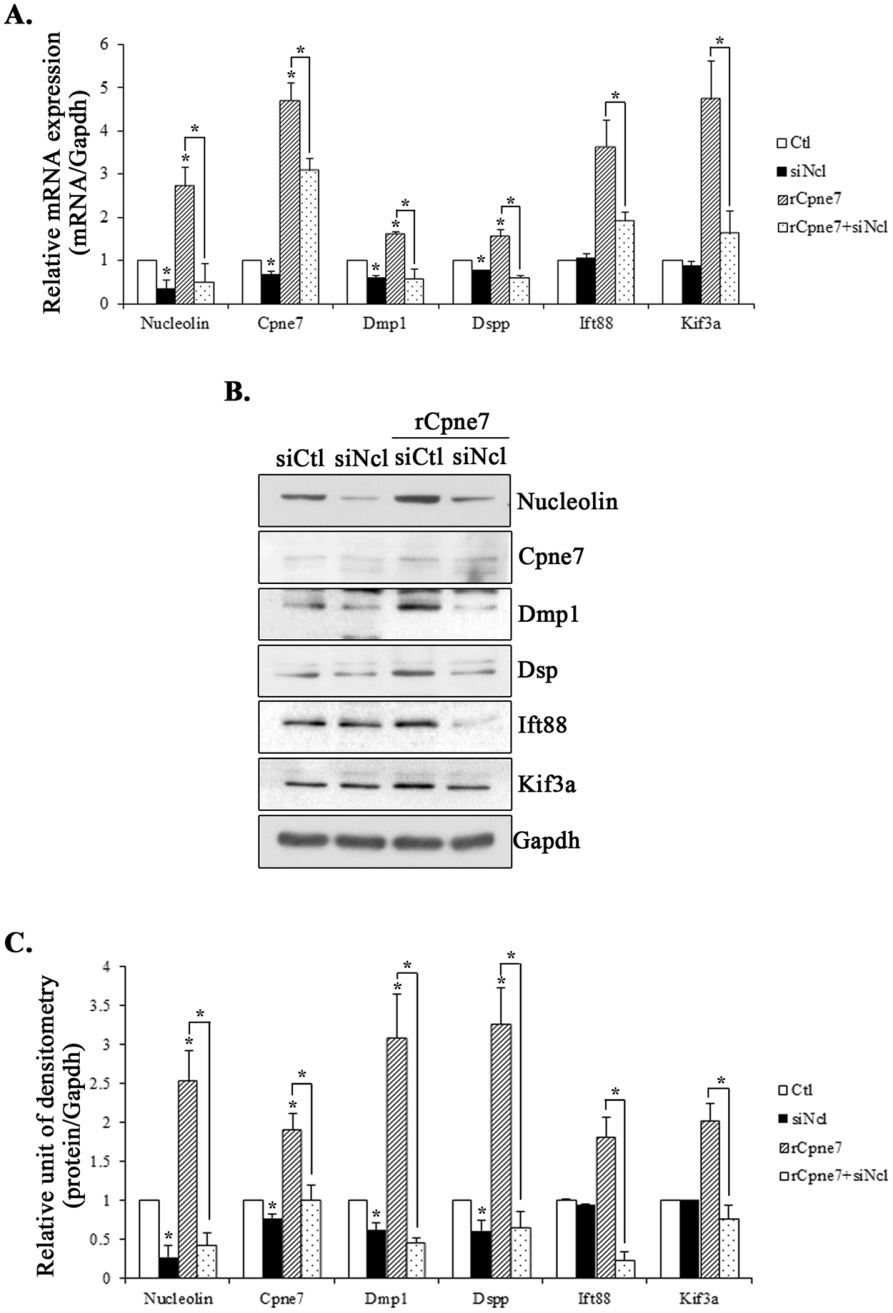
The expression of Cpne7-regulating genes (Dmp1 and Dspp) and cilium components after nucleolin siRNA treatment. The levels of nucleolin, dentin matrix protein 1 (Dmp1) and dentin sialophosphoprotein (Dspp) in nucleolin siRNA-treated MDPC-23 cells were evaluated by quantitative real-time polymerase chain reaction (**A**) and western blotting (**B**) after rCpne7 treatment for 2 days. (**C**) Densitometry analysis of proteins on western blotting are presented according to the formula: (average densitometry assessment protein/average densitometry assessment Gapdh). All values represent the mean ± standard deviation of three independently performed experiments. *P < 0.05 compared with the control. siCtl, control siRNA; siNcl, nucleolin siRNA; Gapdh, glyceraldehyde 3-phosphate dehydrogenase.

Next, we verified whether Cpne7 influenced the expression of cilium components and Dspp through the binding of nucleolin. MDPC-23 cells were treated with rCpne7 in the presence or absence of nucleolin siRNA. Figure 8A–C show that the increase of Dmp1, Dspp, and cilium components, Ift88 and Kif3a, by rCpne7 was dramatically inhibited by nucleolin down-regulation by siRNA. These data indicated that the Cpne7-nucleolin complex stimulated Dspp expression through the regulation of expression of cilium components.

## Discussion

Epithelial-mesenchymal interactions are typical of odontogenesis^29^. Our previous studies suggested that Cpne7 is an epithelial factor that was secreted by preameloblasts, and regulated the differentiation of mesenchymal cells of dental or non-dental origin into odontoblasts^4^. The distribution of the Cpne7 protein in dental cells suggested an important role in epithelial-mesenchymal interactions^4^. Epithelial-mesenchymal interactions during tooth development involve the following steps, 1) interactions involving signalling molecules, 2) transmission of information to adjacent cells, 3) binding of molecules to target cell receptors, 4) activation of a cell cascade in the cytoplasm, 5) entrance into the nucleus to regulate gene expression, 6) expression of new proteins, and 7) a change in the behaviour of target cells. In the present study, we showed that Cpne7 was involved in epithelial-mesenchymal interactions during early odontoblast differentiation.

Nucleolin, with a molecular weight of 105–110 kDa, initially called C23, was originally one of 100 distinct proteins: It comprises 5–10% of the total nucleolar protein in normal rat liver and Novikoff hepatoma ascites cells^30, 31^. Nucleolin was previously thought to be a simple RNA-binding protein involved in the organization of nucleolar chromatin, packaging of the pre-RNA, rDNA transcription and ribosome assembly by shuttling between the nucleus and cytoplasm. Recent studies have further reported that nucleolin is involved in modulating transcriptional processes, cytokinesis, nucleogenesis, signal transduction, apoptosis, induction of chromatin decondensation, and replication^32^. Although ≥90% of the nucleolin is found in the nucleolus, the distribution of nucleolin within the cell is also found in nucleus, cytoplasm, and cell-surface^33^. Cell surface nucleolin is a receptor for various ligands such as midkine, pleiotrophin, P-selectin, ErbB and hepatocyte growth factor (HGF)^19^. More importantly, nucleolin functions as a receptor for internalization of DNPs via lipid rafts^22^, as a receptor for endostatin^20^ and as a receptor for lactoferrin^23^. In the present study, internalization of Cpne7 by preodontoblasts occurred through endocytosis. This endocytic process was mediated by binding of Cpne7 to nucleolin on the cell surface of preodontoblasts. Nucleolin down-regulation by siRNA decreased the endocytosis of Cpne7 in MDPC-23 cells. Together, these results suggested that nucleolin functions as an important receptor for Cpne7.

Nucleolin is also involved in the movement to the nucleus of its ligand as an endocytic receptor. Cines *et al*. reported that nucleolin, interacting with the uPA/uPAR complex, regulated the nuclear translocation of scuPA^34^. Song *et al*. also reported that after the translocation of endostatin by nucleolin from the cell membrane to the early endosome, importin α1β1 recognized the nuclear localization signal of nucleolin, facilitating further transports of the endostatin/nucleolin complex to the nucleus^35^. In the present study, following internalization, Cpne7 was transported to the perinuclear region. Cpne7 in differentiating MDPC-23 cells and hDPCs was present not only in the cytoplasm, but also in the nucleus. The interaction between nucleolin and internalized Cpne7 was detected both in the membrane/cytoplasm and nucleus. Thus, in addition to the internalization and cellular trafficking of Cpne7, nucleolin may also play an important role in nuclear import.

Caveolae-mediated endocytosis is essential for many important signalling pathways and the location of many receptors such as TGFR and the hedgehog receptor^36^. Generally, cell surface caveolae have limited motility and dynamics. However, when triggered by the appropriate ligand, they can be internalized^37^. Acidic non-collagenous protein, Dmp1, was internalized by caveolae in preodontoblasts and hDPCs during tooth development^38^. Nortably, Cpne6 associated with clathrin-coated vesicles in a calcium-dependent manner^9^, and Cpne7 was endocytosed via a caveolae-dependent endocytic pathway when Cpne7 was bound to the cell surface.

Cpne1, 2, 3, 6, and 7 exhibited calcium concentration-dependent translocation to the plasma membrane, and Cpne1, 2, 3, and 7 were also translocated to the nucleus^9^. Cpne7 was localized to the cytoplasm and nucleus of MDPC-23 cells and hDPCs. Moreover, expression of Cpne7 increased during differentiation, especially in the nucleus of differentiating odontoblasts. Our previous results suggested that the Cpne7-nucleolin complex directly regulated transcriptional activation of the Dspp promoter and that it was responsible for Cpne7-mediated Dspp expression during odontoblast differentiation and mineralization^4^. Taken together, these findings suggested that Cpne7 serves as a transcription cofactor/factor during odontoblast differentiation.

Primary cilia, a single and immotile cilia extending from the centriole, were necessary for chemically induced differentiation of human mesenchymal stem cells^28^. During tooth development, the disruption of the function of primary cilia caused dental anomalies, as characterized by missing/supernumerary teeth and dental hypoplasia^39, 40^. Furthermore, primary cilia were required for normal tooth development by integrating Hedgehog and Wnt signalling between the dental epithelium and mesenchyme^41^. These results suggested that primary cilia play important roles in epithelial-mesenchymal interactions. Primary cilia have specialized functions as fundamental structures for mechanical and chemical sensing by individual cells, that transfer signals to the nucleus and cell organelles to induce adequate cellular responses^42^. Odontoblasts are subjected to external stimuli involving the primary cilia as a mediator of mechano-transduction processes concomitantly with mechano-sensitive ion channels^43, 44^. Odontoblasts express cilia components such as tubulin, inversin, rootletin, Ofd1, Bbs4, Bbs6, Alsm1, Kif3a, PC1 and PC2 and cilia are aligned parallel to the dentin walls, with the top part oriented toward the pulp core^15^. Studies using human and mouse dental pulps have reported that primary cilia influenced the terminal differentiation of odontoblasts, and may be functionally connected with tooth pain transmission^15^. Jiang *et al*. reported that inhibition of Kif3a resulted in hDPC disrupted primary cilia formation and/or function, and suggested that Kif3a was an important in the osteoblastic differentiation of hDPCs^45^. Our results showed that the overexpression of Cpne7 resulted in increased formation of primary cilia in hDPCs that affected the expression of the Kif3a and Ift88 ciliary components. Furthermore, the odontogenic gene, Dspp, was significantly down-regulated following Ift88 knockdown in hDPCs and MDPC-23 cells. We therefore suggest that Cpne7 affects differentiation of odontoblast by regulating Dspp expression through ciliogenesis.

**Figure 9:**
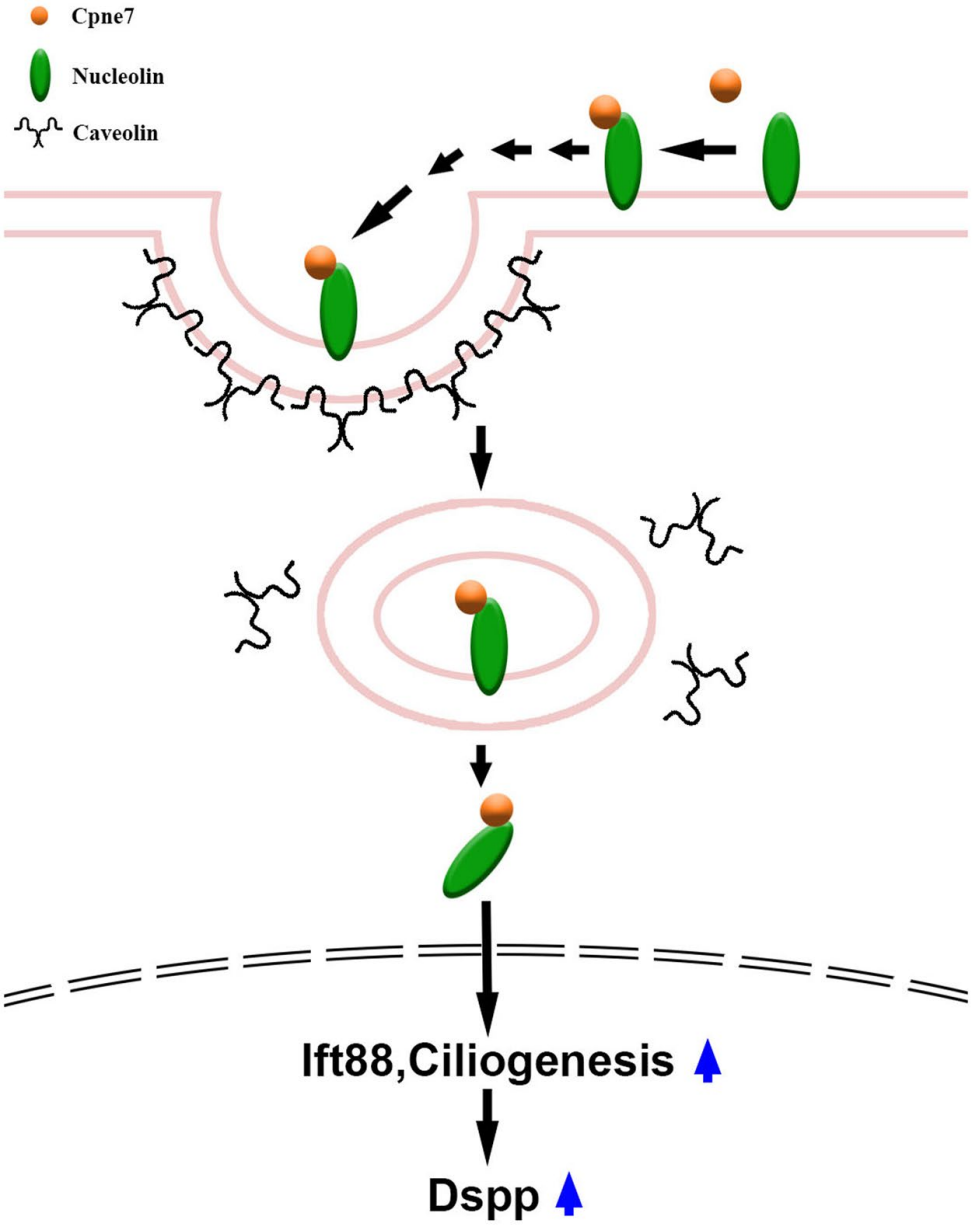
Proposed model for mechanism of action of Cpne7 in odontoblast differentiation. Cpne7 binds to cell surface nucleolin in lipid rafts, and is internalized via caveolae-mediated endocytosis. Cpne7-nucleolin complex is translocated into nucleus of a preodontoblast. Cpne7 increases the formation of primary cilia affecting the expression of Kif3a and Ift88, cilium components. Ift88 promotes up-regulation of Dspp expression.

In our previous study, we showed that Cpne7 enhanced the expression of odontoblast differentiation markers, including Dspp, nestin, and Alp^46^. Specifically, we observed that Cpne7 promoted mineralized nodule formation *in vitro* ^4^. Cpne7 induced the differentiation of non-dental mesenchymal stem cells into odontoblasts and resulted in the formation of dentin-like structures including dentinal tubules *in vivo* ^4^. In this study, the results strongly suggested a mechanism in which Cpne7, an epithelial factor, is internalized into preodontoblasts and transported to the nucleus, which implied possible strategies to regulate odontoblast differentiation (Fig. 9). Cpne7 acted as a ligand and bound to its receptor, nucleolin. The Cpne7-nucleolin complex was then translocated to the nucleus of preodontoblasts. Taken together, we propose that the existence of a Cpne7-nucleolin complex-primary cilia-Dspp pathway during early odontoblast differentiation.

## Methods

### Reagents, antibodies, and plasmids

All reagents used in this study [chlorpromazine, sucrose, methyl-β-cyclodextrin, nystatin, calcium chloride, ethylene glycol tetraacetic acid (EGTA), ethylenediaminetetraacetic acid (EDTA), 4-(2-hydroxyethyl)-1-piperazineethanesulfonic acid (HEPES), potassium chloride, magnesium chloride and dithiothreitol (DTT)] were purchased from Sigma-Aldrich (St. Louis, MO, USA). Rabbit and affinity-purified polyclonal anti-Cpne7, and anti-Dsp antibodies were produced as described previously^46, 47^. Commercial antibodies against Flag (Sigma-Aldrich), nucleolin (Cell Signaling; Danvers, MA, USA), Dmp1 (Abcam; Cambridge, UK), glyceraldehyde 3-phosphate dehydrogenase (Gapdh) (Santa Cruz Biotechnology; Santa Cruz, CA, USA), α-tubulin (Santa Cruz Biotechnology), and Ift-88 (Santa Cruz Biotechnology) were used for Western blot analyses. Expression vectors encoding DDK (Flag)-tagged Cpne7 (NM_153636) and recombinant Cpne7 (NP 705900) were purchased from Origene (Rockville, MD, USA). Control siRNA and nucleolin-targeting siRNA were purchased from Ambion (Carlsbad, CA, USA). Ift88 siRNA was purchased from Santa Cruz Biotechnology.

### Cell culture

Human impacted third molars were collected at the Seoul National University Dental Hospital (Seoul, Republic of Korea), and the experimental protocol was approved by the Institutional Review Board (S-D20140007). Informed consent was obtained from all patients. All methods were performed in accordance with the relevant guidelines and regulations. The human whole pulp cell isolation was described previously^46^. MDPC-23 cells were provided by Dr. J.E. Nor (University of Michigan, Ann Arbor, MI, USA). The hDPCs and MDPC-23 cells were cultured in Dulbecco’s modified Eagle medium (DMEM; Gibco BRL, Carlsbad, CA, USA) supplemented with 10% heat-inactivated foetal bovine serum (FBS; Gibco BRL) and antibiotic-antimycotic reagents (Gibco BRL) at 37 °C in an atmosphere of 5% CO_2_. To induce hPDC and MDPC-23 cell differentiation, 80–90% confluent cells were cultured in DMEM supplemented with 5% FBS, ascorbic acid (50 μg/mL), and β-glycerophosphate (10 mM) for up to 1 week.

### Real-time PCR analysis

Total RNA was extracted from cells with TRIzol reagent according to the manufacturer’s instructions (Invitrogen, Carlsbad, CA, USA). Total RNA (3 μg) was reverse transcribed using Superscript III reverse transcriptase (Invitrogen) and oligo (dT) primers (Invitrogen). One μL of the RT product was PCR amplified using the primer pairs. For real-time PCR, the specific primers for *Cpne7, nucleolin, Dmp1, Dspp, Ift88*, and *Kif3a* were synthesized as listed in Table 1. Real-time PCR was performed on an ABI PRISM 7500 sequence detection system (Applied Biosystems, Carlsbad, CA,USA) using SYBR Green PCR Master Mix (Applied Biosystems) according to the manufacturer’s instructions. PCR conditions were 40 cycles at 95 °C for 1 min, 94 °C for 15 s, and 60 °C for 1 min. All reactions were performed in triplicate, and the PCR product levels were normalized to that of the housekeeping gene, *Gapdh*. Relative changes in gene expression were calculated using the comparative threshold cycle (C_T_) method.

**Table 1:** Oligonucleotide primer sequence used in the real-time PCR.

Gene	Sequence (5′-3′)
*hGAPDH*	F: AGG GCT GCT TTT AAC TCT GGT
	R: CCC CAC TTG ATT TTG GAG GGA
*hCPNE7*	F: GTC TTC ACG GTG GAC TAC TAC T
	R: ATG CGT GTC GTA CAC CTC AAA
*hIFT88*	F: GCA ATC CTA CGA AAC AGT GCC
	R: CAC TGA CCA CCT GCA TTA GC
*hKIF3A*	F: CTC GTC TTC TTC AGG ATT CC
	R: GAG ACT TTC TTT TTT CCC CTT C
*mGapdh*	F: AGG TCG GTG TGA ACG GAT TTG
	R: TGT AGA CCA TGT AGT TGA GGT CA
*mCpne7*	F: CGG GAC CCA TTG ACC AAG TC
	R: CAT ACA CCT CAA ACC GTA GCT TC
*mNucleolin*	F: ACA CCA GCC AAA GTC ATT CC
	R: ATC CTC ATC ACT GTC TTC CTT C
*mDmp1*	F: CAT TCT CCT TGT GTT CCT TTG GG
	R: TGT GGT CAC TAT TTG CCT GTG
*mDspp*	F: GTG AGG ACA AGG ACG AAT CTG A
	R: CAC TAC TGT CAC TGC TGT CAC T
*mIft88*	F: GCA GTG ACAGTG GCC AGA ACA ATA
	R: CAG CCA GGG AGC AGA GAC AAG CAG
*mKif3a*	F: GAA GCC CAA CAA GAG CAT CAG T
	R: CCA GTG GAC GTA GTT TTC AAT CAT

### Western blot analysis

Whole cell lysates of cells were harvested using a lysis buffer consisting of 50 mM Tris–HCl, pH 7.4, 150 mM NaCl, 1% Nonidet P-40, 1 mM EDTA, and 1 mM PMSF supplemented with protease inhibitors (Roche Molecular Biochemicals, Mannheim, Germany). Following centrifugation at 13,000 × g for 30 min, the supernatant was collected for analysis. Protein concentrations were determined using the DC^TM^ protein assay system (Bio-Rad Laboratories, Hercules, CA, USA). Proteins (20 μg) were resolved using 8% or 10% polyacrylamide gel electrophoresis and transferred to a PVDF membrane. The PVDF membrane was blocked with PBST (10 mM phosphate-buffered saline, pH 7.0, and 0.1% Tween-20) buffer containing 5% non-fat dry milk for 1 h at room temperature. The blots were then washed and incubated with the indicated antibodies for 24 h at 4 °C with gentle shaking. Blots were washed three times for 10 min each in PBST, followed by incubation with anti-rabbit or anti-mouse immunoglobulin G conjugated to horseradish peroxidase in PBST for 1 h at room temperature. After washing three times in PBST, the blots were analysed using an enhanced chemi-luminescence reagent (ECL; Santa Cruz Biotechnology) according to the manufacturer’s guidelines. Protein loading was assessed by the expression of Gapdh (1:5000; Santa Cruz Biotechnology). Semi-quantitative measurements were carried out using Image J software (National Institutes of Health, USA).

### Subcellular fractionation

MDPC-23 cells were collected and resuspended in 500 μL ice-cold fractionation buffer [250 mM sucrose, 20mM HEPES; pH 7.4, 10 mM KCl, 1.5 mM MgCl_2_, 1mM EDTA, 1 mM EGTA, 1mM DTT and protease inhibitor cocktail (Roche Molecular Biochemicals)]. The cells were sheared by repeated passage through a 25-gauge needle (10 times). After 20 min, the lysates were centrifuged at 720 × g for 5 min and the pellet was washed once by adding 500 μL of fractionation buffer. The pellet (nuclear fraction) was resuspended in nuclear buffer (RIPA buffer). The supernatant was collected and centrifuged again at 10,000 × g for 60 min. The supernatant was the cytosol/membrane fraction. The pellets (membrane fraction) were lysed by adding 100 μL of RIPA buffer. The nuclear and cytosol/membrane fractions were diluted with an equal volume of 2 × sample buffer and resolved using SDS-PAGE.

### Transient transfection

The hDPC or MDPC-23 cells were seeded into 60 mm culture plates at a density of 1.0 × 10^6^ cells per well. The cells were transiently transfected with DDK (Flag)-tagged Cpne7 using the Metafectene Pro reagent (Biontex, Martinsried, Germany) or with siRNA using the Lipofectamine RNAi MAX reagent (Invitrogen) according to the manufacturer’s instructions.

### Immunoprecipitation (IP)

Cells were transfected with the indicated constructs for 48 h and harvested in IP buffer (50 mM Tris–HCl, pH 7.5, 100 mM NaCl, 1% Nonidet P-40, 1 mM EDTA, and 10% glycerol supplemented with protease inhibitors). The lysates were incubated with the anti-Flag antibody overnight at 4 °C. After incubation for 4 h at 4 °C with A/G-agarose beads (Santa Cruz Biotechnology), the beads were washed three times with IP buffer. Immune complexes were released from the beads by boiling. Following electrophoresis using 8% or 10% SDS-PAGE, the immune-precipitates were analysed by western blot analyses or proteomic analyses.

### In-gel digestion with trypsin and the extraction of peptides

The procedures for the in-gel digestion of protein spots excised from the Coomassie Blue-stained gels were performed as previously described^48^. In brief, the protein spots were excised from the stained gel and cut into pieces. The gel pieces were washed for 1h at room temperature in 25 mM ammonium bicarbonate buffer, pH 7.8, containing 50% (v/v) acetonitrile (ACN). Following the dehydration of gel pieces in a centrifugal vacuum concentrator for 10 min, the gel pieces were rehydrated in 50 ng of sequencing grade trypsin solution (Promega, Madison, WI, USA). After incubation in 25 mM ammonium bicarbonate buffer, pH 7.8, at 37 °C overnight, the tryptic peptides were extracted with 5 uL of 0.5% formic acid containing 50% (v/v) ACN for 40 min with mild sonication. The extracted solution was concentrated using a centrifugal vacuum concentrator. Prior to mass spectrometric analyses, the peptide solution was subjected to a desalting process using a reversed-phase column^49^. In brief, after an equilibration step with 10 μL of 5% (v/v) formic acid, the peptides solution was loaded on the column and washed with 10 μL of 5% (v/v) formic acid. The bound peptides were eluted with 5 μL of 70% ACN containing 5% (v/v) formic acid.

### Identification of proteins by LC-MS/MS

After desalting, the eluted tryptic peptides were separated and analysed using a nano ACQUITY UPLC (Waters, Milford, MA, USA) directly coupled to a Finnigan LCQ DECA iontrap mass spectrometer (Thermo Scientific, Waltham, MA, USA). In brief, the peptides were bound to the ACQUITY UPLC peptide BEH C18 column (1.7 μm size, 130Å pore size, 100 μm × 100 mm) with distilled water containing 0.1% (v/v) formic acid and the bound peptides were eluted with a 40 min gradient of 0–90% (v/v) ACN gradient with 0.1% (v/v) formic acid at a flow rate of 0.4 μL/min. For tandem mass spectrometry, the full mass scan range mode was m/z = 400–2000 Da. After determination of the charge states of an ion on zoom scans, the product ion spectra were acquired in the MS/MS mode with relative collision energy of 55%. The individual spectra from MS/MS were processed using SEQUEST software (Thermo Quest, San Jose, CA, USA) and the generated peak lists were used to query the NCBI database using the MASCOT program (Matrix Science., London, UK). We set the modifications of methionine and cysteine for MS analyses. The tolerance of the peptide mass was 2 Da. The MS/MS ion mass tolerance was 1 Da, allowance of missed cleavage was 1, and the charge states (+1, +2, and +3) were taken into account for data analyses. We considered only significant hits as defined by MASCOT probability analyses.

### Immunofluorescence staining

Cells in Laboratory-Tek chamber slides (Nunc, Rochester, NY, USA) were washed with PBS, fixed with 4% paraformaldehyde in PBS, and permeabilized in PBS containing 0.5% Triton X-100. After washing and blocking, cells were incubated for 1 h with primary (1:200) antibodies in blocking buffer (PBS and 1% bovine serum albumin), followed by the addition of anti-FITC or Cy3-conjugated anti-mouse or rabbit IgG antibodies (1:200; Life Technologies). After washing, cells were visualized using fluorescence microscopy (AX70, Olympus, Tokyo, Japan). The chromosomal DNA in the nucleus was stained using DAPI.

### Statistical analysis

All data were expressed as the mean ± standard deviation from at least three independent experiments. Statistical significance was analysed using one-way ANOVA by the SPSS software version 19.

### Data Availability

The datasets generated and/or analysed during the current study are available from the corresponding author on reasonable request.

## Electronic supplementary material

Supplementary information
