# Supplementary material for: Copine-7 binds to the cell surface receptor, nucleolin, and regulates ciliogenesis and Dspp expression during odontoblast differentiation
Source: Sci Rep. 2017 Sep 12;7:11283. doi: 10.1038/s41598-017-11641-y (PMC5595916; doi:10.1038/s41598-017-11641-y)

Copine-7 binds to the cell surface receptor, nucleolin, and regulates ciliogenesis and Dspp expression during odontoblast differentiation

You-Mi Seo[1†], Su-Jin Park[1†], Hye-Kyung Lee[1], and Joo-Cheol Park[1*]

[1]Department of Oral Histology-Developmental Biology, School of Dentistry and Dental Research Institute, Seoul National University, Seoul, Republic of Korea. †These authors contributed equally to this work.

*To whom Correspondence may be addressed: Joo-Cheol Park, D.D.S., Ph.D., Department of Oral Histology-Developmental Biology, School of Dentistry and Dental Research Institute, Seoul National University, 1, Gwanak-ro, Gwanak-gu, Seoul, 08826, Republic of Korea; Tel.: +82-2-740-8668; Fax: +82-2-763-3613; email: jcapark@snu.ac.kr

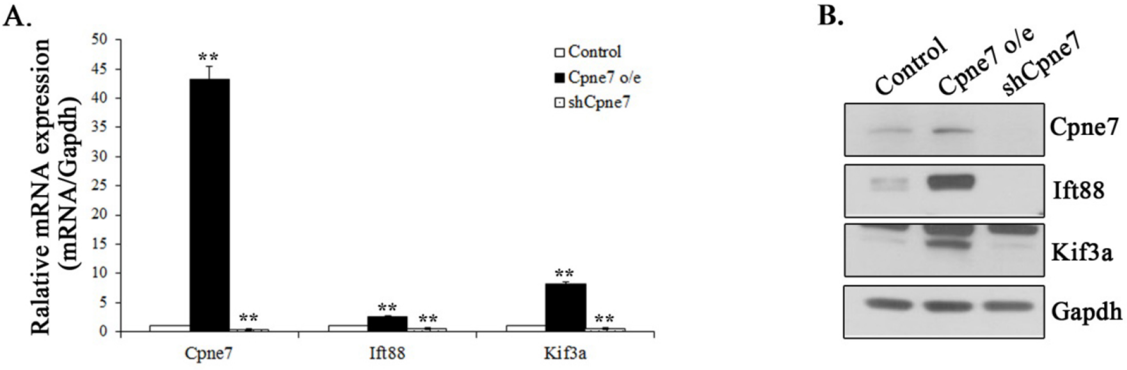

Figure S1. Regulation of the expression of cilium components by Cpne7 in MDPC-23 cells. (A, B) The expression levels of cilium components by Cpne7 were examined by quantitative real-time polymerase chain reaction (A) and western blotting (B) after Cpne7 overexpression or shRNA treatment for 2 days. All values represent the mean ± standard deviation of three independently performed experiments. **P < 0.001, *P < 0.05 compared with control.

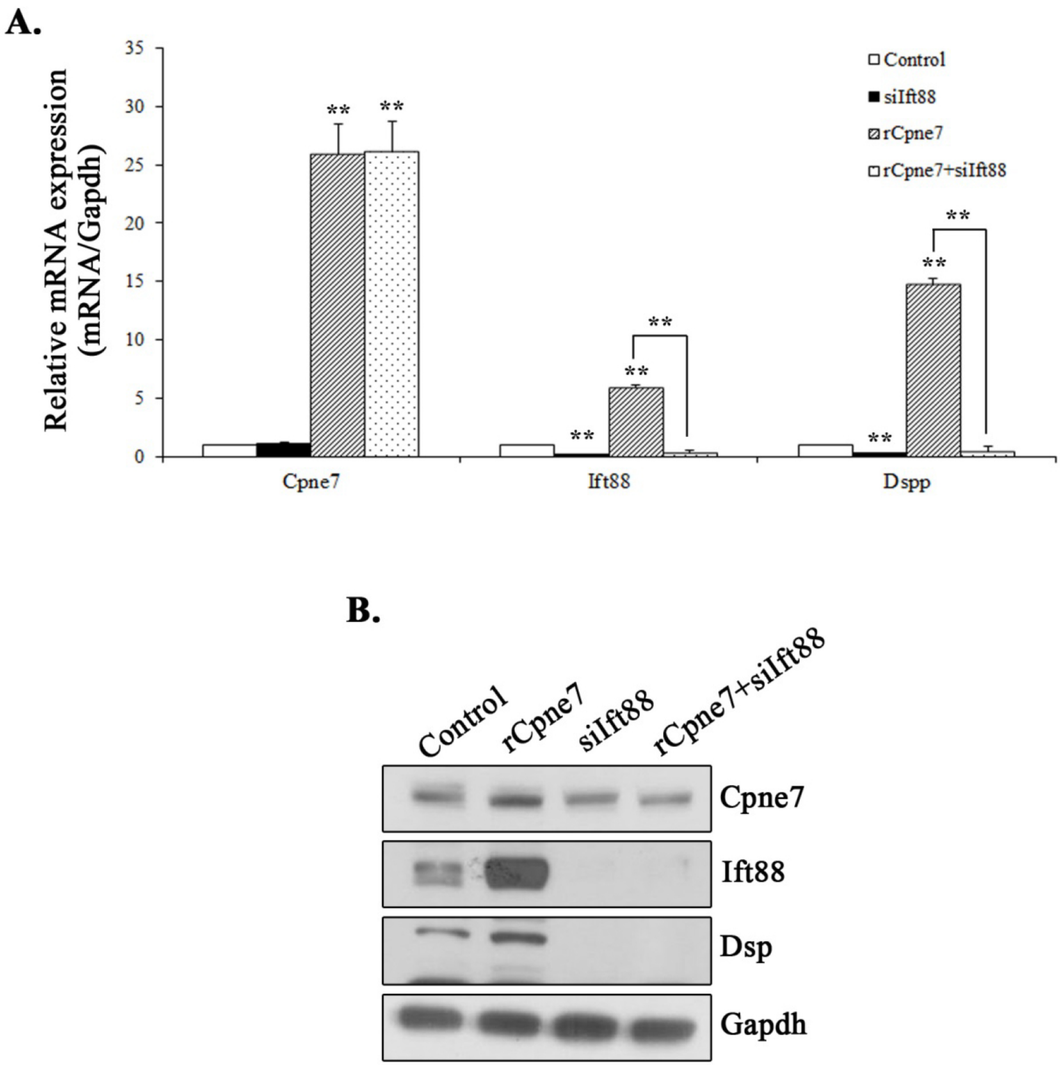

Figure S2. Effect of Ift88 knockdown on Dspp expression in MDPC-23 cells. (A, B) Expression levels of Dspp were examined by the quantitative real-time polymerase chain reaction and western blotting after Ift88 siRNA treatment for 2 days. All values represent the mean ± standard deviation of three independently performed experiments. **P < 0.001 compared with the control.

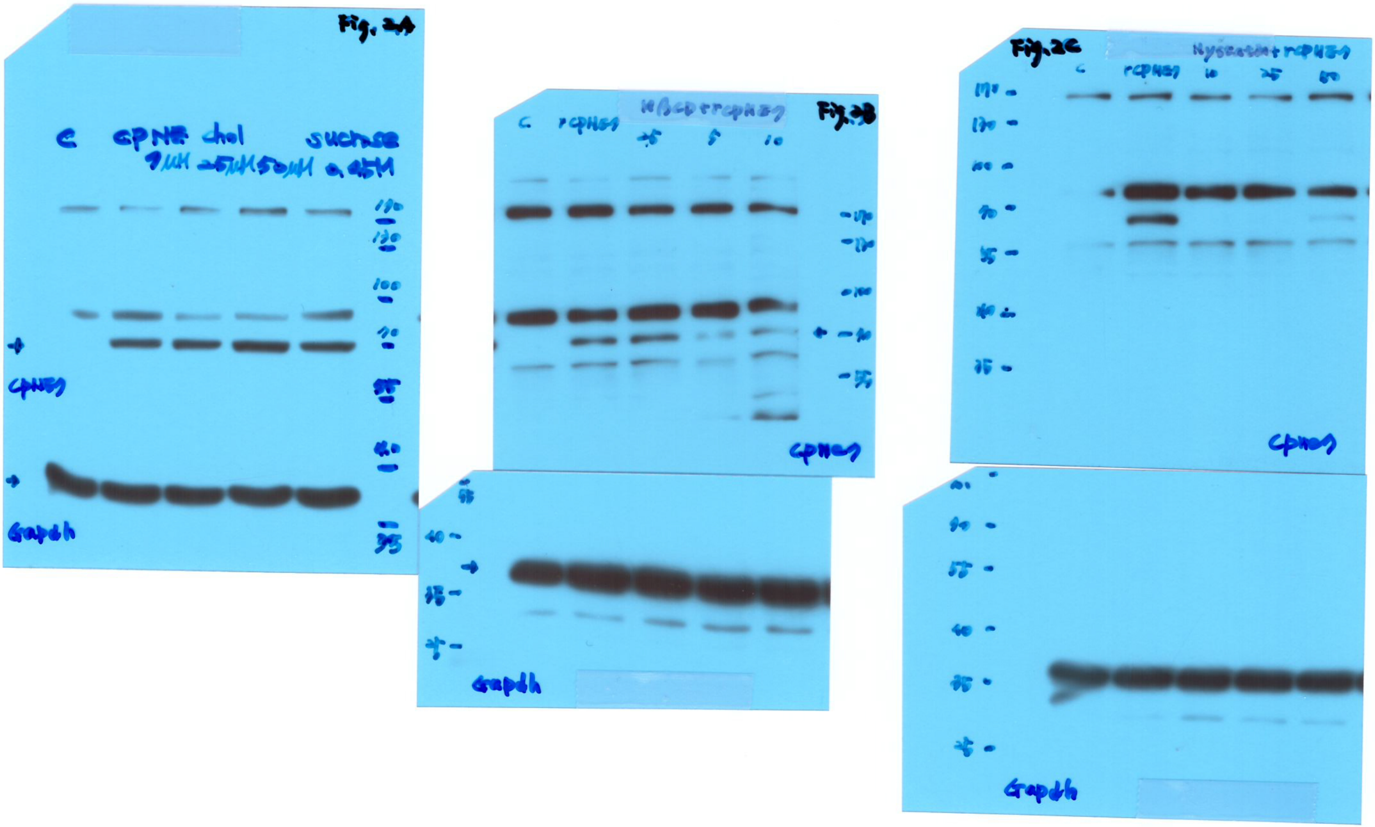
Fig. 2A
C
CPNE chol
sucrose
25uM 50uM
0.25M
170
130
100
70
55
40
35
CPNE9
Gapdh
Fig. 2B
MBCD + rCPNE9
C
rCPNE9
2.5
5
10
170
130
100
70
55
40
35
25
CPNE9
Gapdh
Fig. 2C
Nystatin + rCPNE9
C
rCPNE9
10
25
50
170
130
100
70
55
40
35
CPNE9
Gapdh

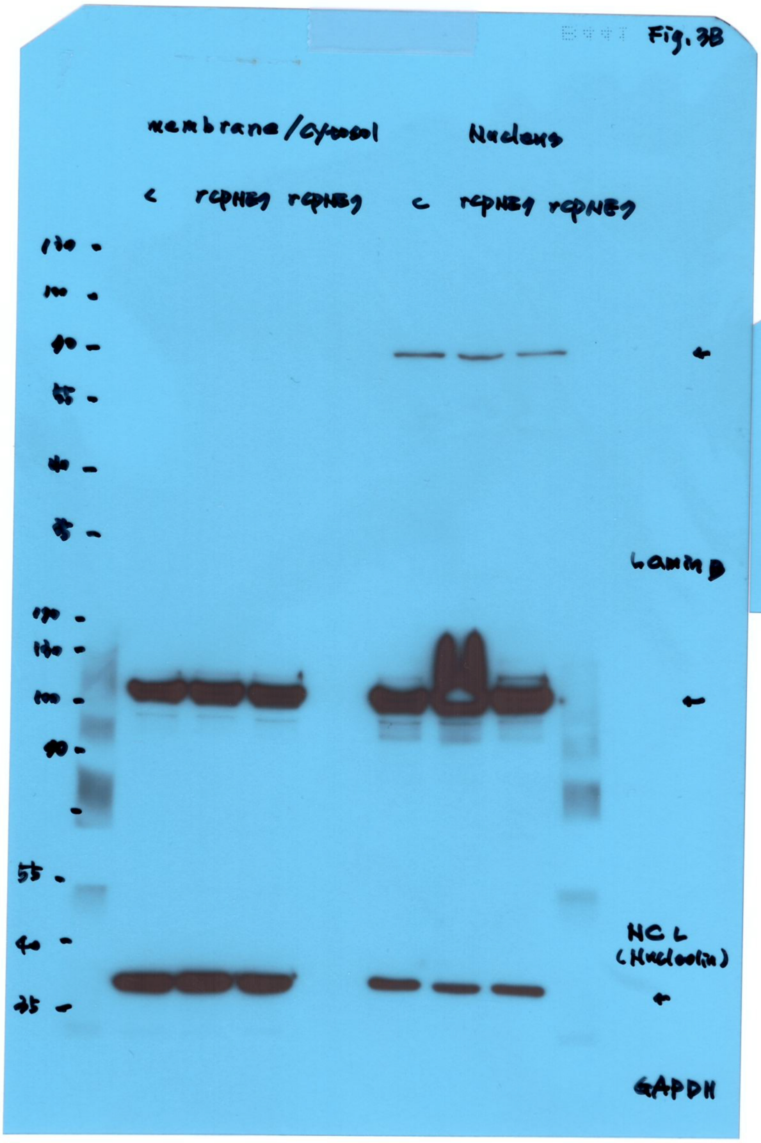
Fig. 3B
membrane/cytosol
Nucleus
c
rcpNE9
rcpNE9
c
rcpNE9
rcpNE9
Lamin B
NCL
(Nucleolin)
GAPDH

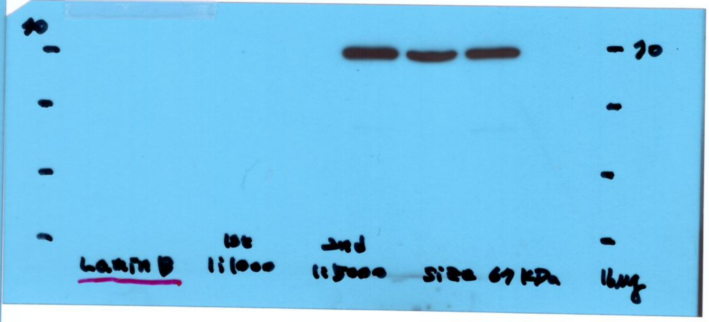
Lamin B
1st
1:1000
2nd
1:5000
size 67 kDa
70

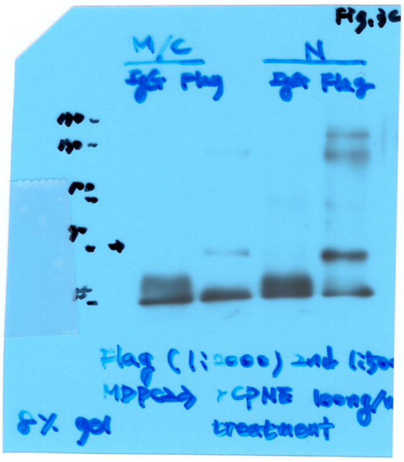
Fig. 3C
M/C
N
IgG
Flag
IgG
Flag
8% gel
treatment

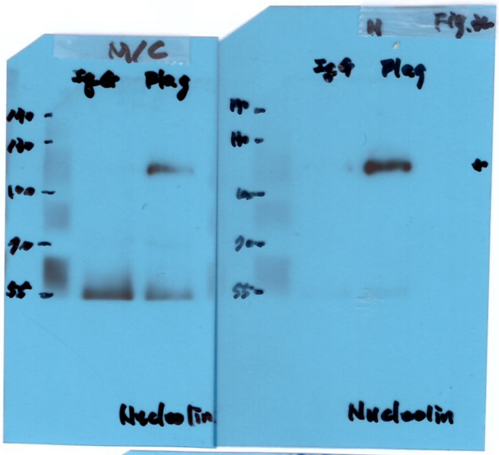
M/C
N
IgG
Flag
IgG
Flag
Nucleolin
Nucleolin

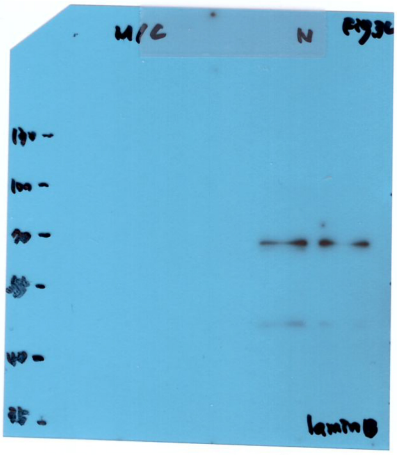
M/C
N
Fig. 3C
lamin B

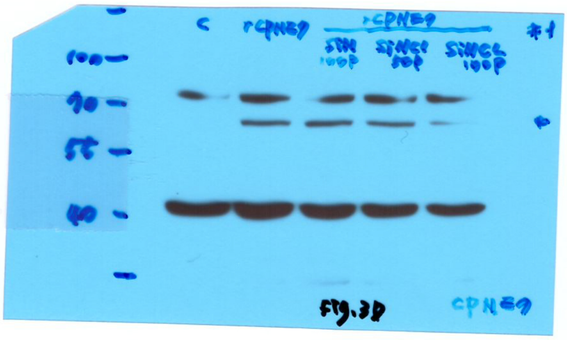
c
rcpNE9
rcpNE9
#1
Fig. 3D
CPNE9

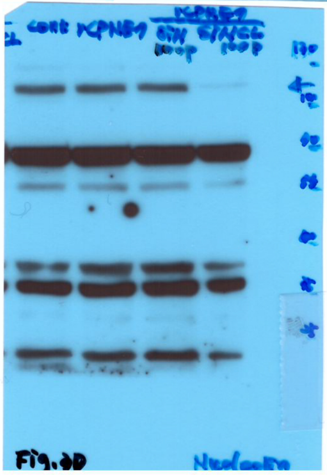
cont
rcpNE9
Fig. 3D
Nucleolin

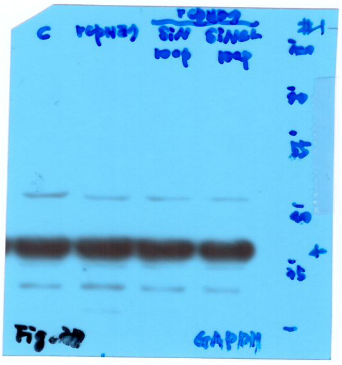
c
rcpNE9
rcpNE9
#1
GAPDH

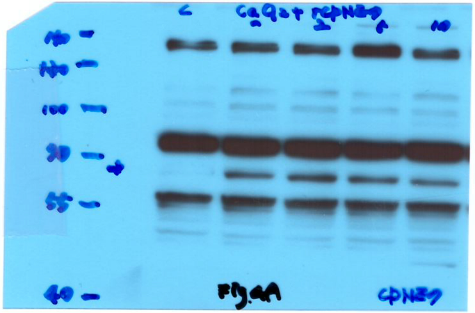
Fig.4A

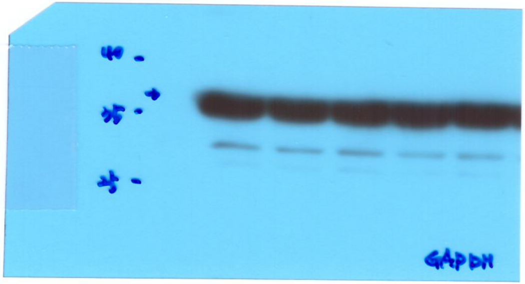
GAPDH

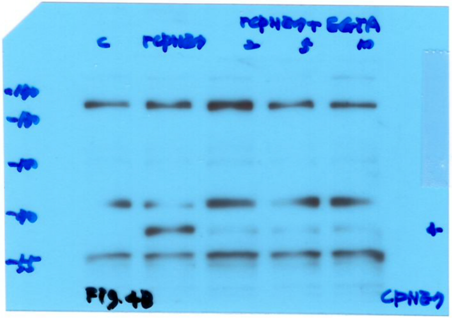
EGTA
Fig.4B

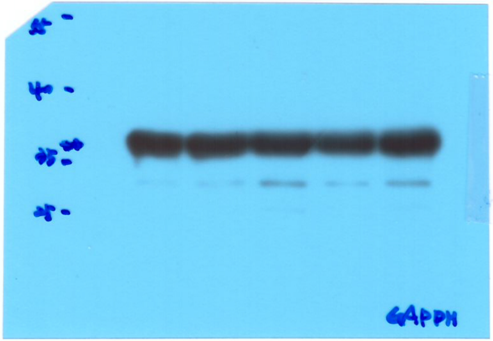
GAPDH

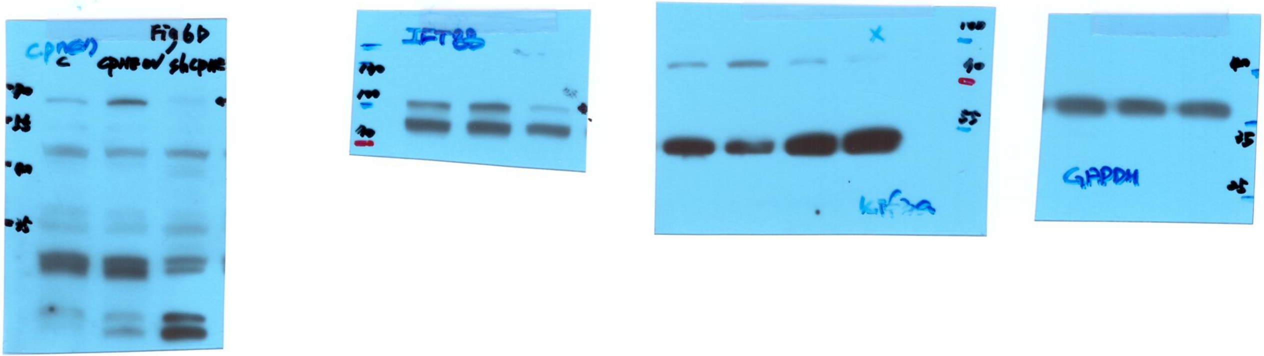
Fig 6D
IFT88
GAPDH

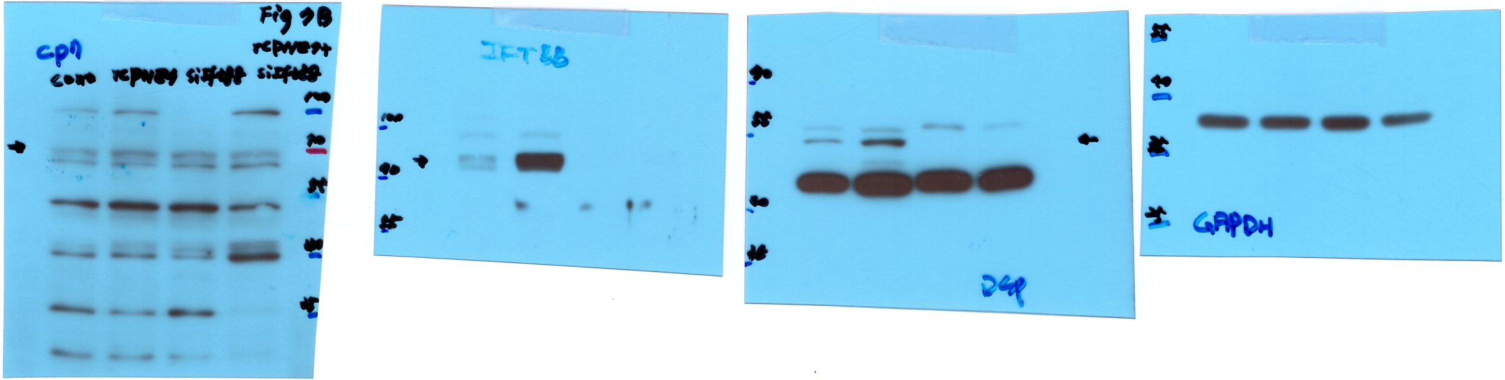
Fig 7B
IFT88
GAPDH

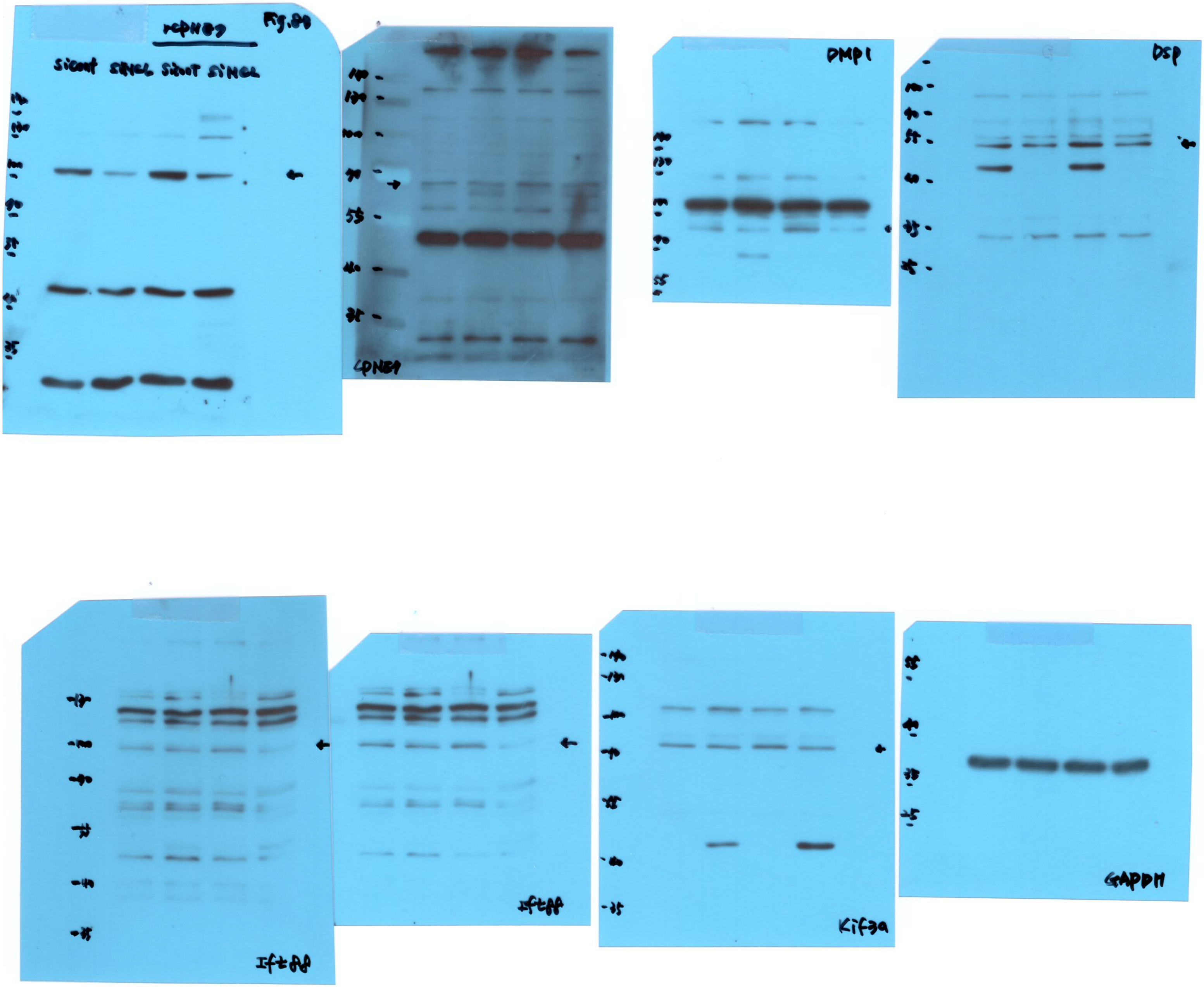
DMP1
DSP
GAPDH
Kif3a

Fig S1.

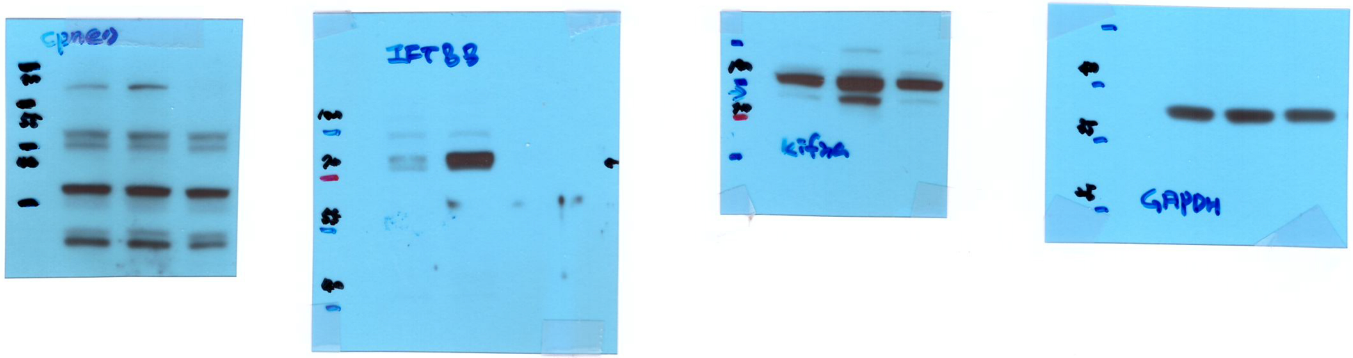

Fig S2.

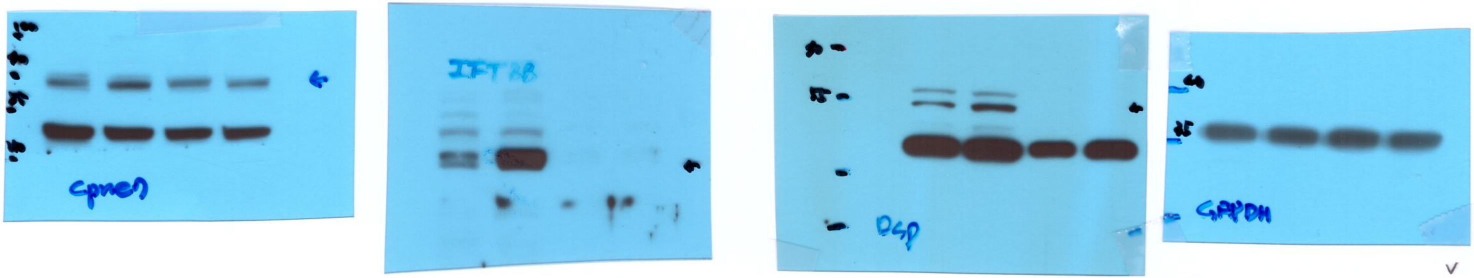

Supplement: Supplementary file 1 — Supplementary information [file 41598_2017_11641_MOESM1_ESM.pdf]
